# 1588. Epidemiology of Chronic High Level (CHL) Epstein-Barr virus (EBV) DNAemia in Pediatric Solid Organ Transplant Recipients (SOTR) at Texas Children’s Hospital (TCH)

**DOI:** 10.1093/ofid/ofac492.111

**Published:** 2022-12-15

**Authors:** Kristen G Valencia Deray, Kathleen Hosek, Daniel Ruderfer, Leanne M Buffaloe, Flor M Munoz, Elizabeth A Moulton, Claire Bocchini

**Affiliations:** Baylor College of Medicine/Texas Children's Hospital, Houston, Texas; Texas Children's Hospital, Houston, Texas; Baylor college of medicine/ Texas Children’s Hospital, Houston, Texas; Baylor College of Medicine, Houston, Texas; Baylor College of Medicine, Houston, Texas; Baylor College of Medicine/Texas Children's Hospital, Houston, Texas; Baylor College of Medicine, Houston, Texas

## Abstract

**Background:**

EBV infections cause significant morbidity and mortality in pediatric SOTR and can be complicated by post-transplant lymphoproliferative disorder (PTLD). Contemporary data on CHL EBV DNAemia and the development of PTLD are limited.

**Methods:**

A retrospective cohort study of patients ≤ 21 years of age who received heart, lung, liver, kidney, or multi-organ transplants at TCH between 2011–2018 was conducted. Primary outcome was CHL EBV DNAemia, defined as peripheral blood lymphocyte EBV values ≥ 500 copies/ug or whole blood EBV values ≥ 10,000 IU/mL for ≥ 6 months. Associations with CHL EBV DNAemia were measured using chi-squared or Fisher exact test and multivariate logistic regression. Time to CHL EBV DNAemia was assessed using Kaplan-Meier method.

**Results:**

Among 687 SOTR (152 heart, 87 lung, 259 liver, 175 kidney, 14 multi-organ), 87 (13%) developed CHL EBV DNAemia; this included 15 (9%) heart, 9 (10%) lung, 59 (22%) liver, 2 (1%) kidney, and 2 (14%) multi-organ recipients. Receiving a heart [OR 2.1, 95% CI (2.1 – 62.4)], lung [7.7, 95% CI (1.5 – 39.3)], liver [OR 31.8, 95% CI (4.9 – 208.7)], or multi-organ [OR 18.3, 95% CI (2.0 – 170)] transplant and being 1–5 years of age [OR 4.0, 95% CI (1.6 – 9.8)] were associated with CHL EBV DNAemia. SOTR transplanted from 2015–2018 were less likely to develop CHL EBV DNAemia than those transplanted from 2011–2014 [OR 0.5, 95% CI (0.3 – 0.8)]. EBV risk status, CMV risk status, gender, and induction therapy were not associated with developing CHL EBV DNAemia.

The median maximum peripheral blood lymphocyte and whole blood EBV values for those with CHL EBV DNAemia were 9475 (993 – 258151) copies/ug and 22093 (564 – 550000) IU/mL, respectively. Organ transplanted (p< 0.01), age (p< 0.01), and EBV risk status (p< 0.01) were associated with time to CHL EBV DNAemia (Figure 1). PTLD occurred in 28 (4%) of SOTR; 14 (50%) had preceding CHL EBV DNAemia (p< 0.01).

Figure 1 Time to CHL EBV DNAemia.

**Conclusion:**

This cohort of pediatric SOTR demonstrates a low prevalence of CHL EBV DNAemia and PTLD. Receiving a heart, lung, liver, or multi-organ transplant and being 1–5 years of age were associated with developing CHL EBV DNAemia. SOTR who developed CHL EBV DNAemia were more likely to develop PTLD, suggesting that further interventions targeting this group may be warranted.

**Disclosures:**

**Flor M. Munoz, MD, MSc**, Gilead: Grant/Research Support **Elizabeth A. Moulton, MD, PhD**, Pfizer: Co-investigator for SARS-CoV-2 pediatric vaccine trials.

